# Prevalence of long COVID decreases for increasing COVID-19 vaccine uptake

**DOI:** 10.1371/journal.pgph.0001917

**Published:** 2023-06-21

**Authors:** Manlio De Domenico

**Affiliations:** 1 Department of Physics and Astronomy “Galileo Galilei”, University of Padova, Padova, Italy; 2 Padua Center for Network Medicine, Padova, Italy; CSIR-Indian Institute of Chemcial Technology, INDIA

## Abstract

Long COVID is a post-COVID-19 condition characterized by persistent symptoms that can develop after SARS-CoV-2 infection. Estimating and comparing its prevalence across countries is difficult, hindering the quantitative assessment of massive vaccination campaigns as a preventive measure. By integrating epidemiological, demographic and vaccination data, we first reconcile the estimates of long COVID prevalence in the U.K. and the U.S., and estimate a 7-fold yearly increase in the global median prevalence between 2020 and 2022. Second, we estimate that vaccines against COVID-19 decrease the prevalence of long COVID among U.S. adults by 20.9% (95% CI: -32.0%, -9.9%) and, from the analysis of 158 countries, by -15.7% (95% CI: -18.0%, -13.4%) among all who had COVID-19. Our population-level analysis complements the current knowledge from patients data and highlights how aggregated data from fully operational epidemic surveillance and monitoring can inform about the potential impact of long COVID on national and global public health in the next future.

## Introduction

There is increasing evidence that post COVID-19 sequelae might occur in individuals following SARS-CoV-2 infection. The definition of post COVID-19 condition by a Delphi consensus requires that symptoms should not find an alternative diagnosis, should persist 3 months after the onset of COVID-19 and should last for at least 2 months [1]. Long COVID, also known as Post-acute sequelae of COVID-19 (PASC), is characterized by a constellation of symptoms ranging from fatigue to shortness of breath and cardiac abnormalities [2–6], which are not limited to the respiratory system [7–9] but might include other systems too, reflecting the onset of metabolic, gastrointestinal, cardiovascular and neurocognitive disorders [10], with risk increasing with the severity of the acute COVID-19 infection [11].

The pathogenesis of long COVID is still unknown, making it difficult to fully characterize, anticipate, diagnose and treat this multisystem disease [12] and risk factors [13]. Consequently, it is difficult to measure its incidence and its prevalence at national and regional scales. Among possible causes, it has been hypothesized that fragments of the virus can persist for months in tissues driving chronic inflammation [14, 15]. Other mechanisms include the triggering of autoimmunity, the dysbiosis of microbiome or virome, and unrepaired tissue damage [16]. It is possible that multiple mechanisms are at work, leading distinct long COVID outcomes: understanding attributes, predictors and risk factors of this disease [17–19] and developing biomarkers [20] might help to stratify patients for a more precise diagnosis and potential treatment (see [21] for the latest review on this topic).

While long COVID is becoming a disease of increasing concern [22–29], here we focus on at least two important questions relevant for policy making that remain to be answered. First, estimates of long COVID prevalence should be reconciled to allow for a better characterization of the underlying epidemiology: in fact, the heterogeneity of the patient cohorts does not allow for a direct comparison of distinct results, which depend on the presence (or absence) of matched control groups, the stratification by immunological status (due to vaccination protocols and natural infections), pre-existing conditions and comorbidities, age and severity of the acute phase of in the infection. The number of clusters corresponding to all combinations of the aforementioned features is so large that it is difficult, in practice, to study all of them. We refer to recent systematic reviews of existing studies for further details [30, 31].

Second, once the prevalence is measured, it is worth quantifying the impact of massive vaccination campaigns on long COVID. While a biological mechanism to support a direct relationship between COVID-19 vaccines and long COVID is still matter of investigation [21, 32], it is plausible to assume that vaccination might induce viral reservoir clearance, according to one of the mechanisms behind the pathogenesis. Recent studies from individual patients data suggest that long COVID outcomes are less likely in vaccinated groups [33, 34].

Understanding the post-viral symptoms of COVID-19 and quantifying the prevalence of long COVID will provide invaluable insights to better understand other existing post-acute infection syndromes [35] and will provide guidelines for policy in future pandemics [36].

## Materials and methods

### Overview of the data sets

The data that support the findings of this study are available from public repositories and/or published papers, without restrictions. A dedicated repository, with the data integrated and processed in this study, is publicly available.

#### Subgroups used for stratification

In the following, we will consider specific subgroups of the population to perform our stratified analysis in a geographic region (e.g., a U.S. state or a whole country). Specifically, we use the following labels:

- **18+**: to indicate the subset of the whole population consisting of adults only
- **18+ who had COVID-19**: to indicate the subset of the whole population consisting only of adults who had COVID-19 in the reference period
- **Same age group**: to indicate the subset of the population referring to a specific age group. The age brackets considered to compare results from the U.K. and the U.S. are 18–29, 30–39, 40–49,50–59, 60–69, 70–79 and 80–99. Another set of age brackets, resampled into narrower bins than the previous ones, consists of 0–5, 6–12, 13–19, 20–29, 30–39, 40–49, 50–59, 60–69, 70–79, 80–89 and 90–99.
- **Same age group who had COVID-19**: to indicate the subset of the population referring to a specific age group, who had COVID-19 in the reference period
- **All who had COVID-19**: to indicate the subset of the whole population who had COVID-19 in the reference period
- **All**: to indicate the whole population

#### Long COVID prevalence in the U.K

The prevalence at national level and for distinct age groups is measured through the Coronavirus (COVID-19) Infection Survey, with respect to the whole population, by the Office for National Statistics (ONS) and made publicly available at this URL [37]. Data refers to self-reported long COVID after infection with the Omicron variant in the UK: we used the 04 August 2022 release and, specifically, the estimated percentage of people living in private households with self-reported long COVID of any duration (four week period ending 02 July 2022). The sample size is *n* = 221, 164 [38]. The data provides also lower and upper 95% confidence limits.

#### Long COVID prevalence in the U.S

The prevalence at state/national level and for distinct age groups is measured through the Household Pulse Survey, with respect to the distinct adult populations, by the Centers for Disease Control and Prevention (CDC) and made publicly available [39]. Data refers to responses to an internet questionnaire, and estimates are weighted to adjust for nonresponse and to match Census Bureau estimates of the population by age, sex, race and ethnicity. We used the reference period corresponding to 29 June—11 July 2022 (*n* = 57, 534). The data provides also lower and upper 95% confidence limits. The source reports that confidence intervals only reflect the potential for sampling error, whereas nonsampling errors (e.g., measurement, coverage, nonresponse and processing errors) can also occur and are more likely for this type of surveys.

#### Cumulative number of infections

The usually reported number of cases refers to confirmed cases. However, it is well known that not all infections are detected, e.g., because it is more difficult to find and test asymptomatic or paucisymptomatic individuals or because contact tracing capacity might be limited. Therefore, we relied on model estimates of the true number of cases over time and, consequently, its cumulative number. Specifically, we used data routinely published by the Institute for Health Metrics and Evaluation (IHME) and the “COVID-19 Data Repository by the Center for Systems Science and Engineering (CSSE) at Johns Hopkins University” [40, 41]. For all countries considered in this study, we have used the IHME data hosted by Our World In Data (OWID), referring to the period 04 February 2020—06 June 2022 [42]. The mean estimate that we consider has been already used in several global public health contexts, e.g. to discuss in detail the weakness of the current global preparedness system [36], discuss policies for the next future [43] and estimate excess mortality [44]. Nevertheless, as any other model, it relies on assumptions and its estimates should be used with caution.

Since we use the IHME estimates, we cannot model counterfactual scenarios, for instance to show what would have happened in the absence of vaccination.

#### Long COVID prevalence estimates at global level

Estimates by country of the number of patients affected by long COVID in 2020 and 2021, regardless of the severity of their symptoms, are obtained from [45]. The data is are based on information for 1,906 community infections and 10,526 hospitalized patients from ten collaborating cohorts, three of which included children, as well as ICD-coded medical record data concerning 1.3 million infections.

#### Vaccination uptake

For the correlation analysis in the U.S. states we have used the data from CDC [46] and hosted by OWID [47]. Specifically we have considered the share of people in each state who received at least one dose of a COVID-19 as of 15 Aug 2022, and the share of people in the state who completed the initial COVID-19 vaccination protocol as of 15 Aug 2022. Note that there can be differences from country to country about the definition of a fully vaccinated individual, depending on the fact that an individual has completed the initial vaccination protocol followed by boosters or not.

For consistency with the data about the long COVID prevalence at global level, and the corresponding analysis, we have used the share of people who completed the initial COVID-19 vaccination protocol by 29 May 2022, provided by OWID, which should reduce any potential bias.

Nevertheless, it should be noted that vaccination uptake, alone, can only be considered as a proxy for population immunity, since natural infection contributes to develop some level of immunity over a limited temporal window.

### Resampling age-stratified data

To overcome some of the limitations of individual, microscopic, analysis due to heterogeneity of cohorts, we use a population-based approach typical of descriptive epidemiology, with a large-scale, macroscopic, analysis to reconcile the estimates of long COVID prevalence across distinct countries and quantify the effectiveness of vaccination to reduce it. Our approach has the main advantage to integrate over all the intervening and characterizing factors of long COVID mentioned above, thus providing a population-level picture that can be used for comparisons across countries.

To map the share of a population or the count (e.g., of confirmed cases) provided for a given age group into different age group we need information about the original reference population, the 1-year age pyramid of the country. For the U.K. and the U.S. we used age pyramids provided for 2022 by the World Population Prospects of the United Nations [48].

For the U.S. total population at the state level, we have used the 2019 Census estimates taken by the United States Census Bureau [49]. Here, we have rescaled the population of each state by the ratio between the U.S. total population in 2022 and the corresponding U.S. state total population in 2019, to have a consistent total population for the subsequent analysis.

For the age-stratified population at the U.S. state level, we have combined the above data with the share of population per distinct age group provided, for 2020, by Kaiser Family Foundation estimates based on the Census Bureau’s March Current Population Survey (CPS: Annual Social and Economic Supplements), 2017-2021.

Indicating by *r*_*A*,*B*|*P*_ the share of a reference population *P* for ages in the group [*A*, *B*], we estimate the population count *n*_*k*_ for age *k* ∈ [*A*, *B*] by

$$\begin{array}{c} n_{k}=r_{A,B|P}P\frac{P_{k}}{\sum_{\ell =A}^{B}P_{\ell }}, \end{array}$$ (1)

and the corresponding share of population by *r*_*k*_ = *n*_*k*_/*P*_*k*_, where *P*_*k*_ indicates the population count for age *k*. Given a distinct age group, e.g., indicated by [*A*′, *B*′], and another reference population *P*′, the corresponding rate is obtained by

$$\begin{array}{c} r_{A^{\prime },B^{\prime }|P^{\prime }}=\frac{\sum_{k=A^{\prime }}^{B^{\prime }}n_{k}}{P^{\prime }}=r_{A,B|P}\frac{P}{P^{\prime }}\frac{\sum_{k=A^{\prime }}^{B^{\prime }}P_{k}}{\sum_{\ell =A}^{B}P_{\ell }}. \end{array}$$ (2)

### Regression models and odds ratio

Since the errors on the estimate are not available, in our linear regression models we account for the presence of confidence intervals on the estimates via weights. Being intervals asymmetric, for each point estimate *x*_*i*_, with bounds [*l*_*i*_, *u*_*i*_], we calculate ${\left[{\left(l_{i}-x_{i}\right)}^{2}+{\left(u_{i}-x_{i}\right)}^{2}\right]}^{-\frac{1}{2}}$, thus giving more importance to more precise estimates.

Given the prevalence of long COVID estimated, with the aforementioned linear model, for the extremal cases of 0% and 100% vaccine uptake, and indicating with *b* ± *ϵ*_*b*_ the estimate on the intercept and with *a* ± *ϵ*_*a*_ the estimate on the slope, all measured in percentages, the odds ratio is given by

$$\begin{array}{c} OR=\frac{b/(100-b)}{\left(a+b)/(100-a-b\right)}. \end{array}$$ (3)

### Visualizing results with density-equalizing maps

Prevalence data are usually visualized via maps which, however, are known to lead to potential misinterpretation of the burden of a disease when sparsely populated large areas dominate densely populated smaller areas. A solution to this problem is provided by density-equalizing maps, also known as cartograms, which reduce the impact of known limitations by applying nonlinear transformations to the political areas (e.g., states in the case of the U.S. or countries in world maps) in order to preserve the proportionality to the variabile of interest. Here, we use the method, based on diffusion, proposed by Gastner and Newman [50].

## Results

We computed the age-stratified prevalence with respect to the same reference populations—the adults, the adults who had confirmed COVID-19 and the whole population of the two countries (Methods)—using data from two national surveys in the U.K. and the U.S., provided by the Office for National Statistics (ONS) and the Centers for Disease Control and Prevention (CDC), respectively.

**Fig 1:**
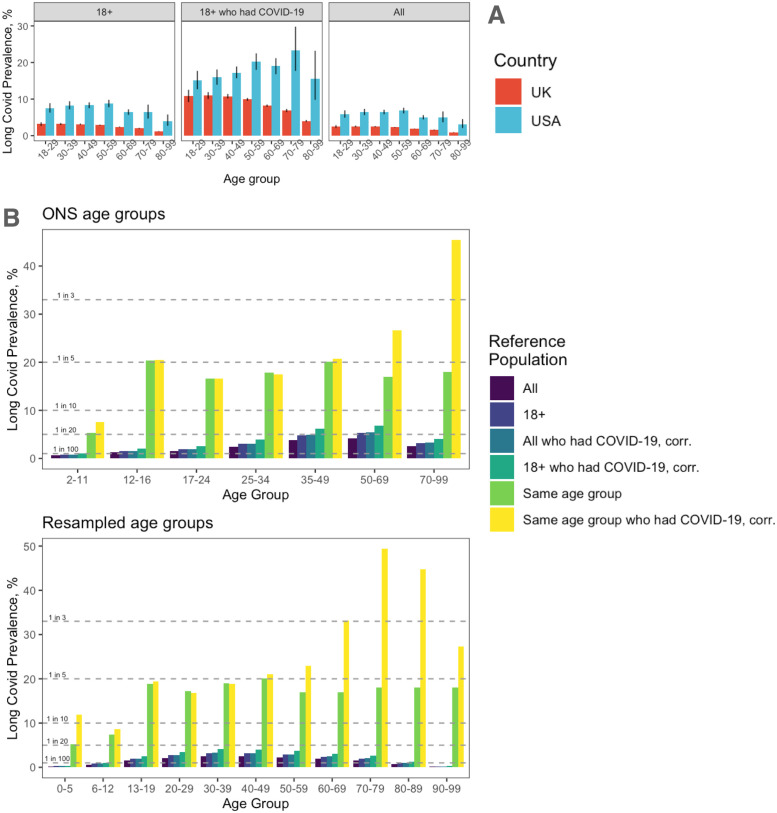
Long COVID prevalence from national surveys. (A) Age-stratified prevalence with respect to age groups defined by CDC and three distinct reference populations (adults, adults who had COVID-19 and the whole population), for the U.K. and the U.S. (B) Age-stratified prevalence in the U.K. with respect to age groups defined by ONS (top) and resampled into narrower bins (bottom). More reference populations are considered and, for the ones involving people who had COVID-19, we corrected the confirmed number of cases by a factor accounting for undetected ones (Methods).

In Fig 1A we show the comparison between the long COVID prevalence in the two countries by using age groups according to CDC, showing that it is systematically higher in the U.S., regardless of the reference population and the age group. For a meaningful comparison, it is of particular interest the population of adults who had COVID-19, where the difference in prevalence is less marked in those aged 18-29 and 30-39. Focusing on the UK only, Fig 1B shows the prevalence for the age groups as provided by the ONS, that we have estimated with respect to distinct reference populations, including the same age group. The original data is provided with respect to the whole population: for the populations related to COVID-19 cases we had to reconstruct the cumulative number of confirmed cases in each age group, and correct it for the estimate of the total number of cases included undetected ones (Methods). People aged 12-16, 17-24, 25-34 and 35-49, show comparable prevalence, stable and close to 1 in 5, with respect to their age group and to who had COVID-19 within their age group. The prevalence is much higher in older age groups. To gain more insights, we have resampled the estimates into narrower age groups: we find that the prevalence reaches 1 in 3 for people aged 60-69 and 1 in 2 for people aged 70-79. For children aged 0-5 and 6-12 the estimated prevalence is about 1 in 10.

**Fig 2:**
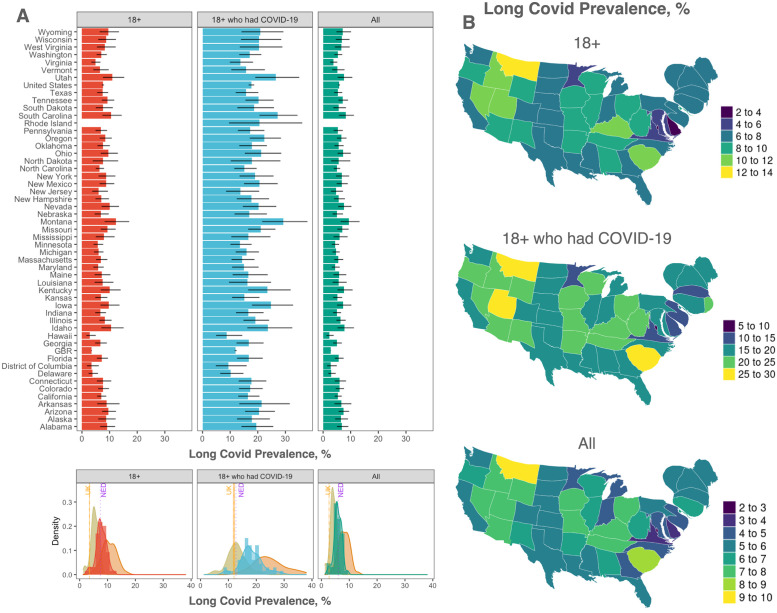
Long COVID prevalence from national surveys. (A) Long COVID prevalence in each U.S. state, the U.S. and the U.K. (labeled as GBR) for the same reference populations in panel A (top) and the distribution of their mean, color-coded consistently with the top panels, and the distribution of the corresponding extremal values—i.e., the 95% lower and upper bounds—(middle). For comparison, we have reported the estimates from the U.K. and the Netherlands [51] as vertical lines. (B) Geographic distribution of the prevalence in the corresponding reference populations by means of density-equalizing maps (Methods). Maps are produced with the software R, package maps, based on Natural Earth free vector and raster map data.

The CDC data also provides information for each U.S. state, although it is not stratified by age. Considering each state as an independent geographic area, we have compared their distribution of the estimated prevalence with respect to distinct reference populations and against the value measured for the U.K. and the Netherlands [51], the latter providing only a mean estimate. Fig 2A shows that the distribution is rather homogeneous, with well identified mean and dispersion, and in good agreement with the estimates from the two European countries. We argue that the signal might be even stronger if measured from the vaccinated population of adults only, although the lack of publicly available data prevented us from further investigations. Fig 2B shows the geographic prevalence of long COVID stratified by age group.

**Fig 3:**
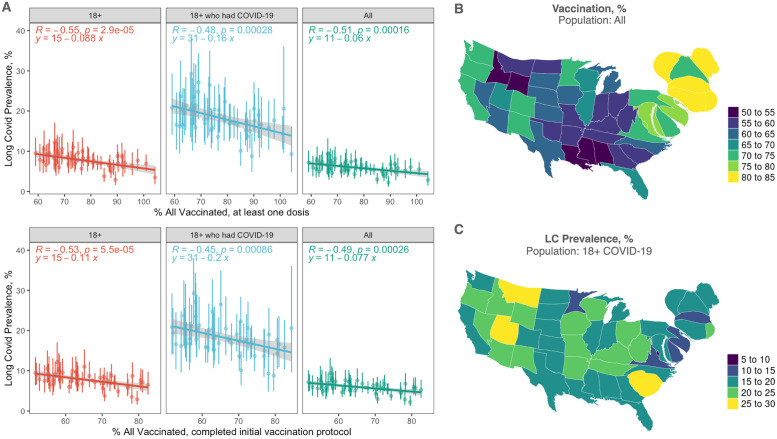
Long COVID prevalence decreases with vaccine uptake in the U.S. (A) Prevalence in U.S. states and the U.S. exhibits a decreasing trend with respect to vaccine uptake, both in the population vaccinated with at least one dose (top) and two doses (bottom), with the largest gap between 100% vaccinated and 100% unvaccinated scenarios observed in the reference population of adults who had COVID-19. The measured correlations are significant (*p* < 10^−3^) in all cases and highlights that the larger the vaccine uptake the lower the prevalence of long covid, with the effect further increasing with more robust vaccination protocol. (B) Global density-equalizing maps highlight the geographic distribution of the vaccine uptake. (C) As in B, for the long COVID prevalence. Maps are produced with the software R, package maps, based on Natural Earth free vector and raster map data.

Stable and consistent estimates of the prevalence allowed us to analyze the impact of COVID-19 vaccination campaigns on long COVID. Fig 3A shows the prevalence against the percentage of the population participating to the vaccination campaign, in each state separately. Fig 3B and 3C shows density-equalizing maps highlighting the geographic distribution of the vaccine uptake and the long COVID prevalence.

We considered two indicators: the population vaccinated with at least one dose and the population who completed the initial vaccination protocol consisting of two doses. Let us focus on the adult population who had COVID-19, which is the one where the effects of long COVID are less smeared out. First, we measure a decreasing trend, highlighting that also from correlation analyses at population level it is possible to measure the signal of the beneficial effects of vaccines. Second, the result is stronger for the population who received two doses: the slope is -20.9% ± 5.5%, suggesting that the difference between a population covered at 100% by the initial vaccination protocol and a population not covered at all leads to a difference of about 21% in developing long covid after an infection. Specifically, the rate for the former is 10.7% (95% CI: 0%–24.0%) and the rate for the latter is 31.6% (95% CI: 24.2%–39.1%). The odds ratio between the unvaccinated and vaccinated scenarios is 3.9 (95% CI: 2.7–5.4).

**Fig 4:**
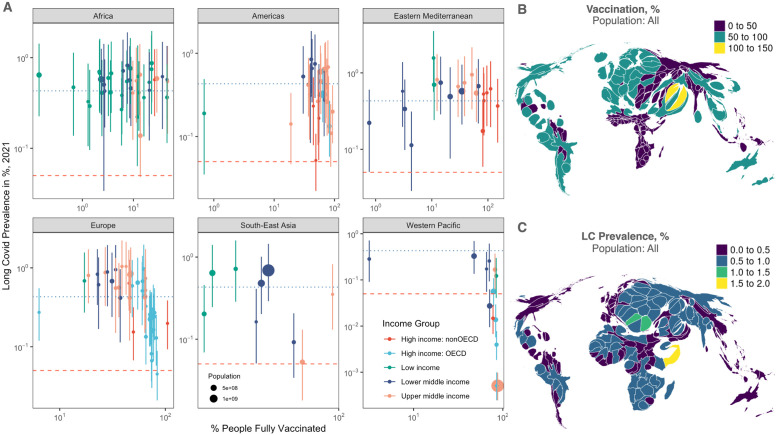
Long COVID prevalence decreases with vaccine uptake, globally. (A) Analysis similar to the one in Fig 4 performed at a global level, using long COVID prevalence estimates for 2021 in the whole population, stratified by WHO region and income group. Dotted blue lines indicate the global median, whereas the dashed red lines indicate the 1 in 2000 limit, used to define a rare disease. (B) Global density-equalizing maps highlight the geographic distribution of the vaccine uptake. (C) As in B, for the long COVID prevalence. Maps are produced with the software R, package maps, based on Natural Earth free vector and raster map data.

We have performed the same analysis at a global level with data from 158 countries, using recent estimates of the prevalence in 2021 [45] and global vaccine uptake rates with respect to the whole population. Fig 4A, where information is further stratified by WHO region and income group, shows patterns similar to the ones observed for the U.S. states, with remarkable differences. Fig 4B and 4C shows density-equalizing maps highlighting the geographic distribution of the vaccine uptake and the long COVID prevalence.

In Africa and in the Eastern Mediterranean regions the correlation signal is not significant due to the low vaccine uptake and, most likely, to the vaccine equity crisis [52–54]. At a global level, the reduction of prevalence is -15.7% (95% CI: -18.0%, -13.4%), which is compatible with the estimate obtained from US for the prevalence among adults only -20.9% (95% CI: -32.0%, -9.9%).

Finally, we use national and global estimates to gain additional insights about the yearly prevalence of long COVID with respect to the whole population. The global median in 2020 was 0.06% (95% CI: 0.02%, 0.13%), while the global median in 2021 was 0.4% (95% CI: 0.1%, 1.0%), corresponding to a 7 fold increase. The estimate for the U.S. in the first half of 2022 is 5.8% (95% CI: 5.5%, 6.1%), a 13 fold increase from the 2021 global median. The estimate for the U.K. is 2.8% (95% CI: 2.7%, 2.8%), a 6 fold increase from the 2021 global median.

## Discussion

Estimating the prevalence of long COVID, the pathological condition that might follow a COVID-19 infection, from ecological data is a difficult task because of the (i) lack of age-stratified data at country level and, overall, (ii) the lack of data for the vast majority of countries. The task is made further difficult by the different reference populations (e.g., all adults above 18 years, adults who had COVID-19, whole population, so forth and so on), which often make difficult to compare the available estimates even across countries releasing the corresponding data. Furthermore, another pressing question highly relevant for global public health concerns the impact of COVID-19 vaccination to reduce the risks of developing long COVID and, consequently to decrease its prevalence at population level.

Concerning the first goal of this study, we used publicly available population data and statistical techniques, we have derived consistent estimates at national and global scales that allowed us to compare, in a meaningful way, the long COVID prevalence across distinct reference populations and age groups. We identified consistent results, with the prevalence in the U.S. being larger than the prevalence in the U.K. Across age groups, long COVID is currently affecting about 1 in 10 children aged 0-12 and 1 in 5 people aged 13-59, with at least a 2 fold prevalence among those ones aged 60-89.

Concerning the second goal of this study, we used our estimates of the prevalence to report a decreasing trend with respect to increasing vaccine uptake, with a reduction of long COVID between 15% and 20%, compatible with recent reports from individual patients data. These results, in the U.S. and global, are in good agreement with estimates from cohorts of individual patients. A recent study, using the US Department of Veterans Affairs national healthcare databases to build a cohort of 33,940 individuals, has estimated a reduction of the risk of long COVID due to vaccinations of about 15%, by analyzing information from 13 million people including contemporary (*n* = 4, 983, 491), historical (*n* = 5, 785, 273) and vaccinated (*n* = 2, 566, 369) controls [33]. Another study, focusing on 9 Italian health care facilities and 2,560 participants, reported that the number of vaccine doses was associated with lower long COVID prevalence: 41.8% (95% CI, 37.0%–46.7%) in unvaccinated patients, 30.0% (95% CI, 6.7%–65.2%) with 1 dose, 17.4% (95% CI, 7.8%–31.4%) with 2 doses, and 16.0% (95% CI, 11.8%–21.0%) with 3 doses [34]. Note that the difference between the case with 3 doses and the unvaccinated rates is about -25.8%, again in good agreement with our estimate from U.S. data.

While results are encouraging, since they provide some evidence that vaccinations are associated with lower prevalence of long COVID, it would be desirable to understand which biological mechanism(s) could support causal relationships. According to the viral remnant hypothesis of long COVID, one of the mechanisms behind the pathogenesis, vaccines could be able to accelerate clearance of SARS-CoV-2 in the human body. Instead, according to another mechanism for pathogenesis, namely the immune/inflammatory hypothesis, vaccines could reduce the amplified immune/inflammatory response to the virus. It is worth remarking that it is possible that multiple mechanisms are at work, although the effective ones are still unknown [31] and require further investigation.

On the one hand, the main advantage of our population-based study is that it naturally integrates over all potential factors (e.g., immunological status, pre-existing conditions, so forth and so on, see [31]) affecting existing estimates, thus providing an effective way to evaluate the effects of vaccines at national level, from an ecological perspective. On the other hand, a limitation of our approach is that it does not allow to quantify the impact of each specific factor contributing to long COVID, as it can be done from individual, microscopic, information. Consequently, there are specific advantages in both ecological and cohort-based analysis, which should be employed together to: (i) characterize the mechanisms leading to post-COVID-19 conditions and develop tailored pharmaceutical therapies; (ii) monitor the prevalence of those conditions at scale, across distinct reference populations, in order to quantify the impact on national health systems and, accordingly, devise adequate public health policies. There are some limitations worth discussing in this context. For instance, the vaccination data does not perfectly reflect the immunity of the population, since infections and breakthrough infections might play a role that cannot be disentangled at an ecological level. Another limitation concerns the use of the IHME estimates, preventing us from modeling counterfactual scenarios—e.g., to show what would have happened in the absence of vaccination—or to perform a sensitivity analysis on its parameters. Therefore, although it is encouraging that our population-level analysis provides estimates that are in good agreement with individual-based results, where it is easier to control for intervening factors, our results should be considered with the adequate caution and cannot prescind from further validation with different models and non-ecological analyses.

Together, our findings suggest that long COVID prevalence and its positive, but not exceptional, response to vaccination have the potential to stress national health systems. The persistent emergence of new SARS-CoV-2 variants such as the sub-lineage BQ.1 in Nigeria (July 2022) and XBB.1.5 in the U.S. (October 2022), followed by localized surges in COVID-19 cases that spread globally within a few months [55, 56], poses important challenges to global public health in the next future. In fact, to characterize the impact on long COVID prevalence of a new SARS-CoV-2 variant it is necessary to wait several months: an amount of time that could have dramatic public health effects in case of sustained community transmission. Consequently, decreasing the risk of infection by reducing community transmission might be an effective approach to control the long COVID prevalence, since it allows to buy the time required to (i) collect new data about the risk of developing post-COVID-19 conditions due to the emerging variant and (ii) further develop pharmaceutical therapies, from antivirals to more effective vaccines. To this aim, data-driven models [55, 57–66] can be used to monitor and control the transmission at national level, as well as to devise effective pro-active measures [67]. For instance, monitoring and controlling air quality [68] can reduce the risk of airborne transmission for any indoor environment [69], but requires clear targets on air changes per hour and air filtration to become an effective public health intervention [70, 71]. Keeping a low infodemic level—i.e., maintaining the circulation of reliable information about the multifaceted aspects of the ongoing pandemic [72–74]—with periodic and adequate communication campaigns, might enhance compliance with public health guidelines [75]. Additionally, coordination among countries is needed to enhance global preparedness [43, 76, 77] to emerging SARS-CoV-2 variants, as well as to novel pathogens with pandemic potential, as the ones due to zoonotic spillover induced by climate change, that according to a recent study, are not going to be unlikely [78].

Complementing vaccination campaigns with potential therapies, as well as further investing on prevention, might reduce the prevalence of long COVID and, consequently, should be considered for public health policies in the next future.
